# The effect of GLP-1RA exenatide on idiopathic intracranial hypertension: a randomized clinical trial

**DOI:** 10.1093/brain/awad003

**Published:** 2023-03-13

**Authors:** James L Mitchell, Hannah S Lyons, Jessica K Walker, Andreas Yiangou, Olivia Grech, Zerin Alimajstorovic, Nigel H Greig, Yazhou Li, Georgios Tsermoulas, Kristian Brock, Susan P Mollan, Alexandra J Sinclair

**Affiliations:** University of Birmingham, Institute of Metabolism and Systems Research, Birmingham, B15 2TT, UK; Department of Neurology, University Hospitals Birmingham NHS Foundation Trust, Birmingham, B15 2GW, UK; Academic Department of Military Rehabilitation, Defence Medical Rehabilitation Centre, Stanford Hall, LE12 5QD, UK; University of Birmingham, Institute of Metabolism and Systems Research, Birmingham, B15 2TT, UK; Department of Neurology, University Hospitals Birmingham NHS Foundation Trust, Birmingham, B15 2GW, UK; University of Birmingham, Institute of Metabolism and Systems Research, Birmingham, B15 2TT, UK; University of Birmingham, Institute of Metabolism and Systems Research, Birmingham, B15 2TT, UK; Department of Neurology, University Hospitals Birmingham NHS Foundation Trust, Birmingham, B15 2GW, UK; University of Birmingham, Institute of Metabolism and Systems Research, Birmingham, B15 2TT, UK; University of Birmingham, Institute of Metabolism and Systems Research, Birmingham, B15 2TT, UK; Drug Design & Development Section, Translational Gerontology Branch, National Institute on Aging, National Institutes of Health, Baltimore, MD 21224, USA; Drug Design & Development Section, Translational Gerontology Branch, National Institute on Aging, National Institutes of Health, Baltimore, MD 21224, USA; University of Birmingham, Institute of Metabolism and Systems Research, Birmingham, B15 2TT, UK; Department of Neurosurgery, University Hospitals Birmingham, Birmingham, B15 2GW, UK; Cancer Research UK Clinical Trials Unit, University of Birmingham, Birmingham, B15 2TT, UK; University of Birmingham, Institute of Metabolism and Systems Research, Birmingham, B15 2TT, UK; Department of Neuro-ophthalmology, University Hospitals Birmingham NHS Foundation Trust, Birmingham, B15 2GW, UK; University of Birmingham, Institute of Metabolism and Systems Research, Birmingham, B15 2TT, UK; Department of Neurology, University Hospitals Birmingham NHS Foundation Trust, Birmingham, B15 2GW, UK

**Keywords:** intracranial pressure, cerebrospinal fluid, glucagon-like peptide 1, idiopathic intracranial hypertension, telemetric monitor

## Abstract

Therapeutics to reduce intracranial pressure are an unmet need. Preclinical data have demonstrated a novel strategy to lower intracranial pressure using glucagon-like peptide-1 (GLP-1) receptor signalling.

Here, we translate these findings into patients by conducting a randomized, placebo-controlled, double-blind trial to assess the effect of exenatide, a GLP-1 receptor agonist, on intracranial pressure in idiopathic intracranial hypertension. Telemetric intracranial pressure catheters enabled long-term intracranial pressure monitoring. The trial enrolled adult women with active idiopathic intracranial hypertension (intracranial pressure >25 cmCSF and papilloedema) who receive subcutaneous exenatide or placebo. The three primary outcome measures were intracranial pressure at 2.5 h, 24 h and 12 weeks and alpha set *a priori* at less than 0.1.

Among the 16 women recruited, 15 completed the study (mean age 28 ± 9, body mass index 38.1 ± 6.2 kg/m^2^, intracranial pressure 30.6 ± 5.1 cmCSF). Exenatide significantly and meaningfully lowered intracranial pressure at 2.5 h −5.7 ± 2.9 cmCSF (*P* = 0.048); 24 h −6.4 ± 2.9 cmCSF (*P* = 0.030); and 12 weeks −5.6 ± 3.0 cmCSF (*P* = 0.058). No serious safety signals were noted.

These data provide confidence to proceed to a phase 3 trial in idiopathic intracranial hypertension and highlight the potential to utilize GLP-1 receptor agonist in other conditions characterized by raised intracranial pressure.

## Teaser

Mitchell *et al*. report that the GLP-1 receptor agonist exenatide significantly reduced intracranial pressure in patients with idiopathic intracranial hypertension, both acutely and after 12 weeks of dosing, in a randomized placebo-controlled trial. The data support moving to a phase 3 trial in idiopathic intracranial hypertension.

## Introduction

Glucagon-like peptide-1 (GLP-1) is a gut neuropeptide secreted by the distal small intestine in response to a meal.^1^ GLP-1 receptor agonists are existing therapeutic agents used in the treatment of diabetes. GLP-1 stimulates glucose-dependent insulin secretion and inhibits glucagon release, thereby lowering blood glucose but not causing hypoglycaemia.^2^ GLP-1 receptor agonists also signal at the hypothalamus to regulate satiety and weight.^3^ This has led to GLP-1 receptor agonists being licensed to promote weight loss in the setting of obesity.^4^

Relevant to this trial is the role of GLP-1 receptor agonists in regulating fluid secretion. GLP-1 receptor agonists have been shown to reduce sodium reabsorption and promote diuresis through actions in the renal proximal tubule.^5,6^ GLP-1 receptors are expressed in the choroid plexus, the predominant CSF-secreting structure in the brain.^7,8^ Our preliminary data have shown that GLP-1 receptor agonism reduces CSF secretion and intracranial pressure (ICP) in an *in vivo* rodent model with elevated ICP.^7^ The reduction in ICP was of a greater magnitude to that observed with the commonly used drugs in idiopathic intracranial hypertension (IIH).^9^

IIH is characterized by increased ICP with no identifiable cause. Recent weight gain is the major risk factor for development of the condition and its occurrence is most commonly observed in women of reproductive age with obesity.^10,11^ Weight loss is disease modifying and if maintained can induce remission.^12,13^ As the incidence of IIH is rising^14–16^ in line with global obesity trends,^17^ targeted treatments are an unmet clinical need.

Visual loss is observed in greater than 90% of those with IIH^18^ with up to 25% suffering marked visual impairment.^19^ Chronic disabling headaches occur in the majority and have adverse impact on quality of life.^20,21^ Cognitive deficits linked to raised ICP have been documented.^22^ Currently, there is no licenced therapy for IIH.^23^ The most commonly used off-label medicine is acetazolamide; other medicines have included topiramate, frusemide, spironolactone and octreotide.^23^ Owing to side effects and treatment failures new therapies are needed. Patient groups have highlighted the importance of prioritizing novel targeted treatments for IIH.^24^

The aim of the IIH:Pressure trial was to translate the preclinical data demonstrating efficacy of GLP-1 receptor signalling to reduce ICP into patients with raised ICP by conducting a randomized, placebo-controlled, double-blind trial in IIH. The trial aimed to evaluate both acute effects on ICP as well as effects over a 3-month time horizon.

## Materials and methods

### Trial design and oversight

The trial was a prospective, randomized, parallel group, placebo-controlled trial in women with active IIH. Patients with a diagnosis of active IIH were identified and recruited from a single tertiary referral hospital (University Hospitals Birmingham NHS Foundation Trust). This study was approved by the West Midlands—Solihull Research Ethics Committee (17/WM/0179) and all subjects provided written informed consent according to Declaration of Helsinki principles. The trial was registered with ISTCRN (12678718).

### Participants

Women aged 18–60 years who met the diagnostic criteria for IIH were recruited.^25^ All had normal brain imaging, including magnetic resonance venography or CT venography (apart from radiological signs of raised ICP). All eligible patients had optic nerve head swelling in at least one eye and ICP >25 cmCSF. Those with significant comorbidities, prior CSF diversion procedures, those currently using GLP-1 receptor agonists or dipeptidyl-peptidase 4 (DPP-4) inhibitors or taking drugs that were thought to reduce ICP were excluded. Those taking drugs that might influence ICP discontinued these at least a month prior to enrolment. Pregnant patients or those planning pregnancy were excluded, urine human choroid gonadotrophin (HCG) was checked at each study visit. Detailed enrolment criteria are provided in Supplementary Table 1.

### Assessments

Following enrolment, a 28-day headache diary was completed [capturing monthly headache days, headache severity (0–10 numerical rating scale), monthly analgesia days]. A telemetric ICP catheter (Raumedic) was implanted prior to the baseline visit through a 4 mm burr hole into the right frontal lobe.

At baseline, medical history, examination (including blood pressure and heart rate) and body mass index [BMI; calculated using the formula: BMI = weight (kg) / height (m)^2^] were recorded and a urine pregnancy test performed. Visual assessments included logarithm of the minimum angle of resolution (logMAR) visual acuity and intraocular pressure as measured with the iCare IC200 (Main-line); perimetric mean deviation (PMD) using the Humphrey visual field analyser [24–2 Swedish Interactive Threshold Algorithm (SITA) standard test pattern using a size III white stimulus]. Papilloedema was confirmed on a dilated slit lamp examination by a neuro-ophthalmologist. It was quantified by spectral domain optical coherence tomography (OCT; Spectralis, Heidelberg Engineering) using the global peripapillary retinal nerve fibre layer (RNFL). Patient-reported outcome measures were assessed [Headache Impact Test-6 (HIT-6)^26^ and 36-item short from survey (SF-36) Rand version].^27^ Assessments were repeated at 12 weeks. Drug compliance was monitored based on the remaining exenatide in the pen injector device.

Blood samples were collected at baseline pre-dose and post-dose at 2.5 h, 6 h, 11 h, 22 h and 24 h, at 2 weeks (pre- and 2.5 h post-dose) and 12 weeks (pre- and 2.5 h post-dose), to evaluate safety blood tests, pharmacokinetics and antidrug antibodies.

ICP was recorded using a transdermal telemetric ICP monitoring system (Raumedic) at baseline, 2.5 h, 24 h and 12 weeks. In the planned exploratory analysis ICP was measured continuously for the first 2.5 h after dosing and then between 24.00 and 07.00 h overnight following the first dose. ICP data were collected at a frequency of 5 Hz and the mean ICP was calculated from each 30 min of continuous ICP monitoring over the first 2.5 h and each hour overnight. ICP was recorded with the MPR-1 monitor (Raumedic) in a standardized supine position as previously described.^28^ The schedule of assessments is detailed in Supplementary Table 2.

### Body composition

Dual energy X-ray absorptiometry (DEXA) was performed using a total-body scanner (QDR 4500; Hologic), as previously described,^29,30^ on a subset of patients. The scans were conducted by a clinical scientist and trained radiographer. In this study no participants were excluded due to having metal prosthetics or implants. Scans were checked for accuracy of fields of measurement. Regional fat mass was analysed as described previously.^29,30^ The precision of total fat mass measures in terms of coefficients of variation (CV) was less than 3%, and for regional fat analyses it was less than 5%. All subjects were analysed on the same DEXA scanner.

### Randomization and study treatment

Participants were randomized in a 1:1 ratio to either active treatment with exenatide (Byetta) or placebo using a computer-generated randomization list generated by the Birmingham Clinical Trials Unit. Treatment allocation was blinded to patient and investigators. A double check of allocation was performed by an unblinded nurse and pharmacist. The first dose was a loading dose of subcutaneous exenatide 20 μg or equivalent volume of subcutaneous 0.9% saline placebo. Subjects were then dosed for 12 weeks (self-administered at home) with either subcutaneous exenatide 10 μg or equivalent volume of placebo twice daily. There was no provision for access to treatment after the study concluded.

### Blood analysis

The following fasted blood tests were processed at the hospital laboratory: creatinine (μmol/l), alanine aminotransferase (ALT; IU/l), high-density lipoproteins (HDL; mmol/l), total cholesterol (mmol/l), triglycerides (TG; mmol/l), haemoglobin A1c (HbA1C; mmol/mol). Samples not analysed immediately were centrifuged (10 min at 1500*g* at 4°C) aliquoted and stored at −80°C. All samples only underwent a single freeze–thaw cycle.

### Fasting insulin and homeostasis model assessment of insulin resistance

Fasting insulin (Mercodia) was measured using a commercially available assay, according to the manufacturer’s instructions. Homeostasis model assessment of insulin resistance (HOMA2-IR) was calculated using the program HOMA calculator v2.2.3.^31^

### Pharmacokinetics

Exenatide concentration was evaluated by ELISA. ELISA was performed on serum samples from seven patients receiving the active drug. Serum was collected at 10 time points: baseline, 2.5 h, 6 h, 11 h, 22 h and 24 h, Week 2 at baseline and 2.5 h post-dose, Week 12 at baseline and 2.5 h post-dose. An exenatide fluorescent ELISA (Phoenix Pharmaceuticals Inc) was used. This was a competitive enzyme immunoassay wherein the primary antibody is competitively bound by either a biotinylated peptide or the targeted peptide in samples. All samples were run in triplicate. The assay was performed according to the manufacturer’s protocol.^32^

### Anti-exendin-4 antibody levels in serum

In the light of reports of the development of anti-exenatide antibody in human studies administering exenatide in type 2 diabetes mellitus^33,34^ (with anti-exenatide titres peaking between 6 and 22 weeks), as well as their presence in preclinical studies,^32,35^ we investigated whether such antibodies developed in our clinical study. As the trial lasted for a period of 12 weeks, we evaluated serum anti-exendin-4 antibody levels in 16 patients at baseline (prior to exenatide treatment), and at Weeks 2 and 12 of the trial by employing a previously developed sandwich ELISA.^35^ All Weeks 2 and 12 serum samples were obtained before exenatide administration for the day, and serial dilutions of each serum samples were used in the ELISA. A brief protocol for the ELISA is as follows: plates were coated with exenatide (exendin-4; 2 µg/ml concentration in coating buffer; AnaSpec Inc) at 4°C overnight. Thereafter, following blocking and washing steps, standards (mouse monoclonal anti-exendin-4 antibody; Abcam ab23407) and unknown samples with serial dilutions were added to the plate and incubated at room temperature for 1 h. After washing, biotinylated-exendin-4 (2 µg/ml concentration; AnaSpec Inc) was added and followed by washing and SA-HRP detection (KPL). The titres of the anti-exenatide antibody within the samples were then estimated by serial dilution of the serum (to a maximum dilution of 1:125).

### Outcomes measure

The primary outcome was ICP at 2.5 h, 24 h and 12 weeks post-drug administration. ICP was recorded with p-Tel telemetric ICP catheter and MPR-1 reader (Raumedic). ICP was recorded continuously for 30-min periods at specified time points in a standardized supine position. For the first 2.5 h post-dosing, mean ICP was calculated from each 30 min of continuous ICP monitoring. For the overnight recording the mean ICP was calculated from each hour of continuous ICP monitoring. ICP was sampled at 5 Hz. Recordings were downloaded and analysed in Dataview version 1.2 (Raumedic). ICP was recorded in mmHg (conversion factor to cmCSF was 1.36).

Secondary outcomes included: monthly headache days, headache severity and monthly analgesia days, logMAR visual acuity measured using the Early Treatment Diabetic Retinopathy Study (ETDRS) charts; PMD using Humphrey 24–2 SITA central threshold automated perimetry; BMI and health-related quality of life (measured by SF-36 and HIT-6). Evaluations were at baseline 2.5 h, 24 h and 12 weeks (with additional blood sampling at 2 weeks).

### Adverse event reporting

Adverse events were recorded as was drug compliance (unused medication in the injector pens documented).

### Sample size calculation

In a study of 25 patients, Sinclair *et al*.^13^ showed that the cross-sectional sample standard deviation (SD) of ICP is 4.9–5.1 cmCSF, measured at baseline and immediately before and after a longitudinal intervention (low-energy diet). There are very few trials in IIH and the minimal clinically important change for lumbar puncture pressure is not established and may vary with individual patients. Seeking significance at least *α* < 0.1 and power at least 80% using equal group sizes, a total sample size of 14 patients was required, i.e. seven patients randomized to receive active treatment and a further seven to receive control. This calculation assumed an effect size of 6.5 cmCSF with an SD of 5.1 cmCSF, the upper end of the range observed previously. Allowing for 10% drop-out, the proposed recruitment was eight patients per arm and 16 patients in total.

### Statistical analyses

All primary analyses (primary and secondary outcomes including safety outcomes) were evaluated by intention-to-treat (ITT) analysis. Analysis was completed on received data, with every effort made to follow-up participants to minimize potential for bias. Final analyses were conducted after the final visit of the final patient of the main trial once the data had been cleaned and locked, then unblinded. No imputation of missing data was conducted. The analysis of visual data included data from the most affected eye at baseline as defined by PMD, analysis of intraocular pressure was performed on the mean average of both eyes. Statistical analysis was performed in R v4.0.0 (R Foundation for Statistical Computing, Vienna, Austria). Data were reported as means and SD [with median and interquartile range (IQR) for non-normal data], and standard error (SE) and 95% CI where appropriate. Hierarchical linear regression models were used to analyse repeated measures of the primary and secondary outcomes and to estimate differences adjusted for baseline values. In these models, population-level effects (also known as fixed effects) comprised the intercept, time as a factor variable and the two-way interaction of treatment arm and time as a factor variable to model changing treatment effects over time. Group-level effects (also known as random effects) comprised patient-level adjustments to the intercept. The threshold for statistical significance was pre-specified at 0.1.

### Data availability

Anonymized individual participant data will be made available along with the trial protocol and statistical analysis plan. Proposals should be made to the corresponding author and will be reviewed by the Data Sharing Committee in discussion with the Chief Investigator. A formal Data Sharing Agreement may be required between respective organizations once release of the data is approved and before data can be released.

## Results

### Patients

**Figure 1:**
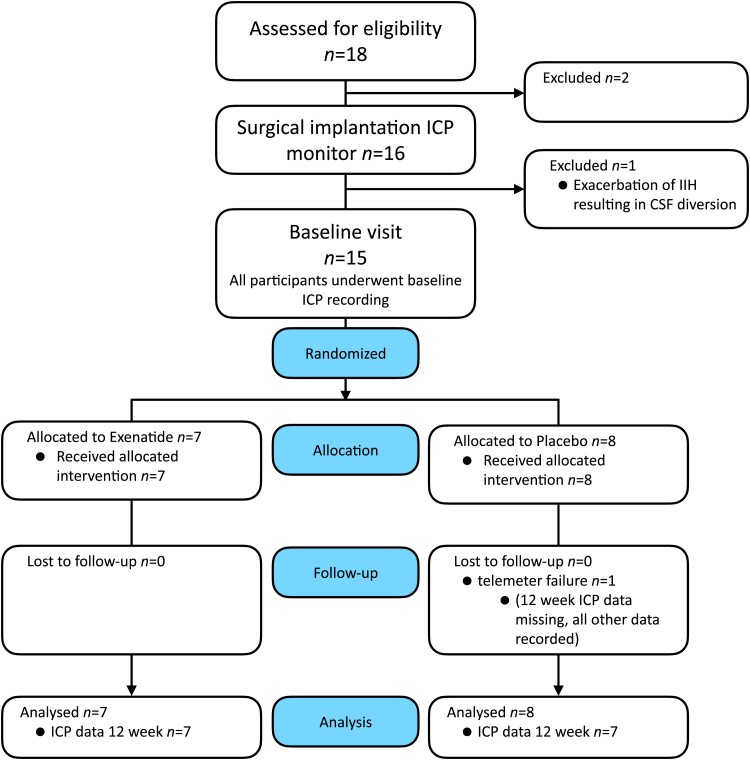
**Consort diagram.** Consort diagram describing the numbers and disposition of study subjects.

Between 1 November 2017 and 17 September 2018, 18 participants were screened, 16 enrolled and 15 randomly assigned to either the exenatide group (*n* = 7) or the placebo group (*n* = 8) (Fig. 1).

### Adherence to the protocol

One participant was withdrawn before randomization due to disease progression requiring urgent CSF shunting (Fig. 1). All randomized patients completed the 12-week duration of the trial; however, one patient experienced telemeter failure prior to Week 12 and therefore ICP data were not recorded; all other data for Week 12 were collected for this patient. There was full adherence to protocol with no crossover and no significant protocol deviation, except blood tests for pharmacokinetics, which were missed on four occasions.

### Demographics and baseline characteristics

Baseline characteristics confined a cohort of patients with active IIH (Supplementary Table 3). Age, BMI and ICP at baseline were well-matched between groups. Median [IQR time from surgical implantation of ICP monitor to baseline visit was 10 days (16.5); Supplementary Table 3]. At baseline there was a significant difference in monthly headache days between arms, exenatide 21.6 (5.2) [mean (SD)] and placebo 10.3 (8.5); there was also a significant difference in PMD, exenatide −0.6 (1.0) dB [mean (SD)] and placebo −2.7 (1.9) dB (Supplementary Table 4).

### Primary outcome measure

**Figure 2:**
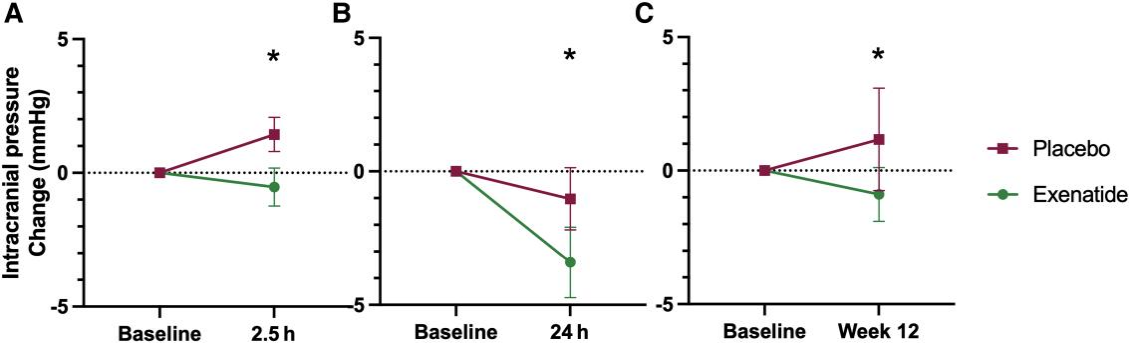
**Primary outcomes.** Mean change (SEM) of ICP in (**A**) ICP at 2.5 h; (**B**) ICP at 24 h; (**C**) ICP at 12 weeks. *P < 0.1 (significant level set at *P* < 0.1). Diurnal variability in ICP reflected in changes in ICP readings at different times of day observed in the placebo arm.

The primary clinical outcome was the difference in ICP between exenatide and placebo, as measured by telemetric ICP monitoring at 2.5 h, 24 h and 12 weeks. The difference in ICP between arms at 2.5 h was −4.2 (2.1) [mean (SD) mmHg], *P* = 0.048 and at 24 h was −4.7 (2.1), *P* = 0.030. The effect was sustained at 12 weeks with an ICP difference of −4.1 (2.2), *P* = 0.058 (Table 1 and Fig. 2). This was equivalent to 5.7 (2.9) cmCSF [mean (SD)] at 2.5 h, 6.4 (2.9) cmCSF at 24 h and 5.6 (3.9) cmCSF at 12 weeks.

**Table 1:** Primary outcome measures

	ICP at baseline, mean (SD)	ICP at time point, mean (SD)	Difference in ICP baseline to time point, mean (SD); 95%CI, *P*	Difference in ICP between arms at time point, mean (SE); 95%CI, *P*
**ICP 2.5 h (mmHg)**				
Exenatide (*n* = 7)	22.3 (3.6)	21.8 (3.4)	−0.5 (1.9); (−2.3, 1.2), *P* = 0.485	−4.2 (2.1); (−8.4, 0.0), *P* = 0.048
Placebo (*n* = 8)	24.6 (4.1)	26.0 (3.4)	1.4 (1.8); (−0.1, 2.9), *P* = 0.060	
**ICP 24 h (mmHg)**				
Exenatide (*n* = 7)	22.3 (3.6)	18.9 (5.3)	−3.4 (3.5); (−6.6, −0.2), *P* = 0.042	−4.7 (2.1); (−8.8, −0.5), *P* = 0.030
Placebo (*n* = 8)	24.6 (4.1)	23.5 (4.5)	−1.0 (3.3); (−3.8, 1.7), *P* = 0.406	
**ICP 12 weeks (mmHg)**				
Exenatide (*n* = 7)	22.3 (3.6)	21.4 (4.0)	−0.9 (2.7); (−3.3, 1.6), *P* = 0.410	**−4.1 (2.2); (−8.4, 0.1), *P* = 0.058**
Placebo (*n* = 7)	24.6 (4.1)	26.0 (4.4)	1.2 (5.1); (−3.5, 5.8), *P* = 0.565	

### Secondary clinical outcomes

**Figure 3:**
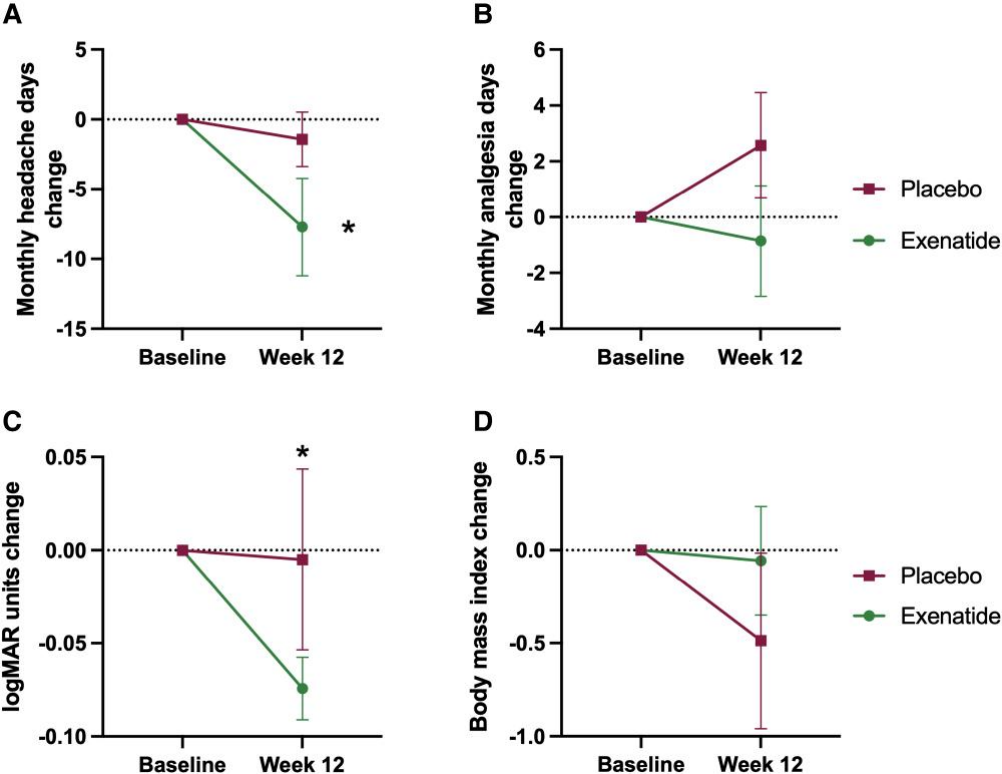
**Monthly headache days and analgesia, visual acuity and BMI.** Mean change (SEM) at 12 weeks of (**A**) monthly headache days, (**B**) monthly analgesia days, (**C**) visual acuity measured by logMAR and (**D**) body mass index (BMI). *P < 0.1 (significant level set at *P* < 0.1).

The key secondary outcome was monthly headache days, which reduced significantly in the exenatide arm, −7.7 (9.2) days [mean (SD)], *P* = 0.069 compared to the placebo arm −1.5 (4.8) days, *P* = 0.404 (Fig. 3), although there was no significant difference between arms at 12 weeks. Changes in monthly analgesia days showed a trend to improvement in the exenatide arm, but not in the placebo arm (Fig. 3 and Supplementary Table 4). There was no significant change in headache severity (Supplementary Table 5). Headache disability measured by HIT-6 was significantly higher in the exenatide arm at baseline 62.9 (3.2) versus 55.8 (6.9) [mean (SD)], *P* = 0.041, and there was no significant change over the course of the trial (Fig. 3 and Supplementary Table 6).

### Vision

Visual acuity significantly improved in the exenatide arm compared to the placebo arm −0.1 (0.05) logMar units, *P* = 0.036. There was no significant change in PMD, difference between arms 1.0 (0.8) dB, *P* = 0.188. OCT RNFL did not change significantly between arms, −40.2 (47.2) µm, *P* = 0.396 (Fig. 3 and Supplementary Table 4).

### Intraocular pressure

There was no significant change in intraocular pressure, difference between arms −0.1 (1.1), *P* = 0.910 (Supplementary Table 4).

### Quality of life

Analysis of quality of life, using the SF-36, showed no significant changes in either the physical or mental component scores (Supplementary Table 4).

### Body mass index

BMI in the placebo arm baseline was 38.6 (4.7) kg/m^2^ and 38.1(4.9) kg/m^2^ at 12 weeks, whereas in the exenatide arm baseline was 37.6 (7.9) kg/m^2^ and 37.5 (7.4) kg/m^2^ at 12 weeks. There was no significant difference in BMI between arms at 12 weeks, *P* = 0.854 (Fig. 3 and Supplementary Table 4).

### Safety blood test results

No significant changes were seen in the safety monitoring blood tests (Supplementary Table 7).

### Adverse events

Twelve adverse events were reported during the trial. Eight adverse events occurred in the exenatide arm, seven of which were nausea related to exenatide initiation. Four adverse events occurred in the placebo arm. One unrelated serious adverse event, thyrotoxicosis, was reported in the placebo arm. No patients withdrew due to adverse events (Supplementary Table 8). All participants remained compliant with assigned treatment as monitored by returned medication.

### Anti-exenatide antibodies

No anti-exenatide antibodies were detected in samples collected at baseline. In total, two of the seven patients on exenatide developed antibodies by Week 12. At Week 2 one patient had antibodies present (> 0.1 µg/ml) and these were also present at 12 weeks (> 0.1 µg/ml). A further patient was noted to have antibodies at 12 weeks (>1.0 µg/ml).

### Drug concentrations measurements

The two patients with anti-exenatide antibodies at 12 weeks had higher (3- and 6-fold higher) than predicted exenatide concentrations at 12 weeks and were excluded from the 12 weeks data analysis. At baseline, Week 2 and Week 12, there was a sharp increase in exenatide concentrations in patient serum at 2.5 h following subcutaneous administration of exenatide. This was followed by a sharp decline in peptide concentrations at 6 h. The mean exenatide concentrations at 2.5 hours are higher at baseline than at Week 2 and Week 12 (575.4, 380.7 and 205.4 pg/ml, respectively), which is expected as 20 μg of exenatide was administered at baseline, compared to 10 μg administered at Weeks 2 and 12 (Supplementary Table 9 and Supplementary Fig. 1). The number of participants was insufficient for meaningful analysis of the relationship between pharmacokinetics and efficacy.

### Glucose and insulin

No hypoglycaemia was encountered during the trial. There was no significant difference in fasted glucose and fasted insulin between the two trial arms at 12 weeks (Supplementary Table 10 and Supplementary Fig. 2).

### Vital signs

Blood pressure and heart rate were stable during the trial, mean arterial pressure was lower at 12 weeks in the exenatide group compared to placebo, −6.7 mmHg (3.6), *P* = 0.088, but there was no significant change within arms between baseline and 12 weeks (Supplementary Table 4).

### *Post hoc* analysis: exploratory ICP

**Figure 4:**
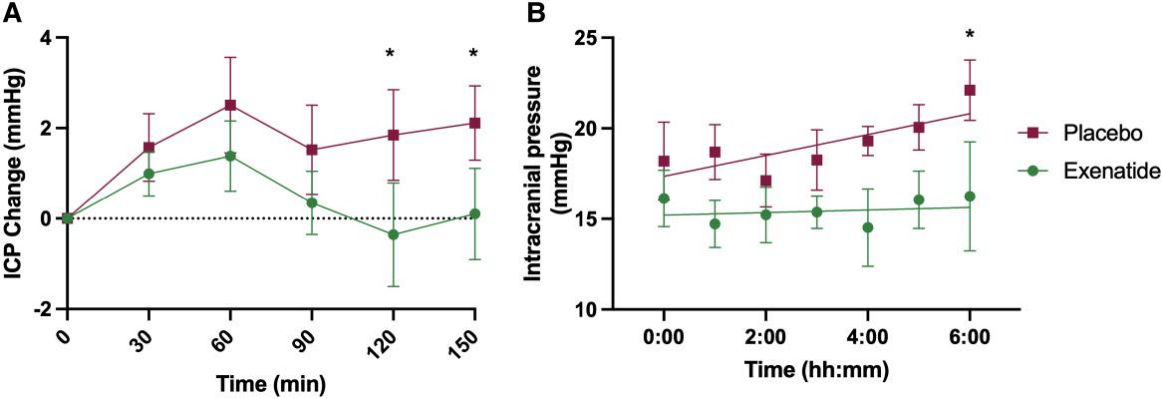
**First 2.5 h, overnight ICP.** Mean ICP (SEM) by arm measured (**A**) continuously over 2.5 h after dose and (**B**) hourly overnight. *P < 0.1 (significant level set at *P* < 0.1).

We looked in more detail at the changes in ICP in the first 2.5 h following drug administration. ICP was significantly lower in recordings taken over time points 90–120 min [mean (SD): −4.6 (2.1) mmHg, *P* = 0.028] and 120–150 min [mean (SD) −4.2 (2.1) mmHg, *P* = 0.042] (Fig. 4A and Supplementary Table 11).

ICP was then measured overnight between 24.00 and 07.00 h on the first day of dosing (Fig. 4B and Supplementary Table 12). ICP is observed to rise progressively overnight in IIH.^36^ In the placebo arm the ICP rose overnight as expected by 3.1 (4.1) mmHg, *P* = 0.13. In the exenatide arm, the overnight rise in ICP was supressed to 0.1 (3.2) mmHg, *P* = 0.97. There was a significant difference in the ICP measurement between the exenatide and placebo arms at the last reading of the night [06.00–07.00 h; mean (SD) ICP difference between arms was 5.0 (2.5) mmHg; equivalent to 6.8 (3.4) cm CSF; *P* = 0.04].

### DEXA

We determined that IIH patients had a similar fat mass, lean mass and android–gynoid ratio in those treated with exenatide as compared to those on placebo with no significant differences detected at 12 weeks (Supplementary Table 13).

## Discussion

This is the first randomized controlled trial comparing the efficacy of the GLP-1 receptor agonist exenatide, with placebo to reduce ICP in patients with active IIH. We have found a significant and clinically meaningful reduction in ICP in the treatment arm as compared to the placebo arm both acutely and after 12 weeks of dosing. The drug was safe and well-tolerated. This trial establishes the successful translation of preclinical data into humans and is the first to demonstrate a new drug treatment for IIH that significantly reduces ICP in IIH.

The hallmark of IIH is raised ICP, which causes headaches and visual loss (through compression of the optic nerve and papilloedema).^10,21,37^ Therefore, to evaluate the efficacy of exenatide the primary outcome for this trial was chosen as ICP.^38^ A significant reduction in ICP was seen acutely at 2.5 h of −4.2 mmHg (equivalent to −5.7 cmCSF), which corresponds to peak exenatide serum levels. The reduction in ICP was also noted at 24 h [reduction of 4.7 mmHg (equivalent to 6.4 cmCSF); Table 1 and Fig. 2]. There are no studies that have evaluated other drugs (used off-label) to treat IIH that have shown ICP reduction within the first 24 h of administration. IIH patients can deteriorate rapidly (over days)^39^ and hence a drug with rapid onset of action is clinically advantageous for IIH. A drug treatment that can rapidly reduce ICP is also of potential relevance for other conditions characterized by raised ICP, such as following traumatic brain injury and stroke.

A significant reduction in ICP was also noted after prolonged dosing at 12 weeks (−4.1 mmHg equivalent to −5.6 cmCSF; Table 1 and Fig. 2), demonstrating durability of dosing. The minimal clinically important change in ICP in those with IIH has yet to be determined.^38^ There have been four previous randomized control trials in IIH^12,40–42^ and one prospective crossover trial.^13^ Four of these have used ICP as an outcome measure, with the magnitude of treatment effect between trial arms ranging from 5.9 cmCSF (acetazolamide and diet),^41^ −6.0 (1.8) (by bariatric surgery)^12^ and −6.2 cmCSF (through low-calorie diet).^13^ The efficacy in these studies is akin to what we have observed following treatment with exenatide for 3 months (−5.6 cmCSF).

Headache is a key disabling feature for IIH patients and listed in the top 10 priority areas for research by patient groups.^24^ Headache is also a key determinant for the significantly reduced quality of life in IIH.^20^ In this trial, while not powered to evaluate secondary outcome measures, there was a significant reduction in monthly headache days between baseline and 12 weeks among those patients taking exenatide (−7.7 days); those in the placebo arm did not have any significant improvement (−1.5 days; Fig. 4 and Supplementary Table 4). A reduction in monthly headache of 1.5–2 days is regarded as meaningful in chronic migraine randomized controlled trials.^43–46^ Improvements in headache in IIH have been shown in other trials to occur due to reduction in ICP.^21^ Headache outcomes may show more benefit over a longer trial duration, as during the relatively short time horizon of this trial other factors may also have contributed to headache such as medication overuse and opiate use, which occur in approximately one-third of IIH patients.^43^

There was a significant improvement in visual acuity between the trial arms at 12 weeks (Fig. 3 and Supplementary Table 4). In this trial, the magnitude of change (five letters) was equivalent to a line on the visual acuity chart. In other ocular diseases a change of more than five letters would have >90% probability of being a real change.^47^ Changes in visual acuity of five letters have been noted previously in a prospective crossover cohort study evaluating a weight loss intervention where there was a reduction of ICP by −6.2 cmCSF.^13^ It is likely that the improvement in visual acuity found here may be related to resolution of the hyperopic shift associated with raised ICP and papilloedema.

Weight loss has been shown to reduce ICP in IIH.^12,13^ In this study, there was no significant reduction in body weight at 12 weeks in the treatment arm, which implies that the reduction in ICP was likely to have been mediated by a reduction in CSF secretion, the mechanism demonstrated in preclinical studies, rather than through weight loss.^7^ Additionally, the significant reduction in ICP noted at the early time points of 2.5 h and 24 h would have been too premature to be modulated by weight loss. GLP-1 receptor agonists such as exenatide can increase satiety and promote weight loss, but this is generally in a context of a concurrent low-calorie diet.^48^

Exenatide is widely used to treat type 2 diabetes and has an established long-term safety record (licensed in 2005).^49,50^ In this trial exenatide was safe and well-tolerated, with no withdrawals due to drug adverse effects. This is relevant as the drug predominantly used off-label for IIH, acetazolamide, can be poorly tolerated. In a previous randomized controlled trial 48% of patients withdrew over a 3-month period, due to adverse effects.^40^ Nausea is a known side effect of GLP-1 receptor agonists,^49,50^ and was predicted to occur in those IIH patients taking exenatide. In this trial seven patients experienced nausea related to exenatide, but this settled over the first week. The nausea may, in part, be explained by the exenatide pharmacokinetic profile with high peak levels noted twice a day with twice daily Byetta dosing.^51^ Our pharmacokinetic data illustrated highest serum levels at 2.5 h post-dose, in keeping with the established literature on Byetta. Anti-drug antibodies were detected in two participants. The clinical relevance of this is not determined; however, anti-drug antibodies have not been shown to impact drug effects in diabetes.^34^ IIH is known to be a disease of insulin resistance^52^ and therefore exenatide’s insulin-sensitizing effects may have additional benefits in this population.

This is the first trial, to our knowledge, to use telemetric intracranial monitoring to measure ICP in IIH. Patient input into the trial design advocated for telemetric ICP monitoring over multiple lumbar punctures to facilitate data validity and reduce patient discomfort from multiple lumbar punctures. This has permitted detailed characterization of ICP changes and the ability to measure ICP for prolonged periods over several weeks without further invasive procedures.^53^ This technology has led to the accurate demonstration of ICP lowering both with single dosing and with repeated dosing. The intracranial telemetric catheters were safe, with only one failing to register by 12 weeks.

This study has several limitations. As an early phase study, it was powered to the primary end points of ICP reduction with single and repeated dosing. Thus, the study was underpowered to detect differences in patient-centred outcomes and secondary clinical end points such as headache and visual field perimetry. Powering the study for those end points would have required a large increase in number of participants; however, telemetric ICP monitors would not be feasible in large trial cohort. No stratification was used at randomization and groups were unmatched for baseline headache days and visual field perimetry. Additionally, patients with minimal visual field defects were not excluded as in prior studies, thus making the study more clinically representative, but visual effects more difficult to detect due to the ceiling effect of the measurement. With the main side effect of exenatide being nausea, patients could have been unmasked; however, the primary end point was a physiological measure, which should minimize this risk.

The results of this study may have wide reaching implications for many other diseases of raised ICP as the drug does not target the specific pathogenesis of IIH, but CSF secretion. Given the tolerability of exenatide and its direct and early effect on ICP, it may be beneficial in other conditions such as hydrocephalus, traumatic brain injury, raised ICP in stroke and meningitis and space flight–associated neuro-ocular syndrome. In addition, GLP-1 receptor agonists have been shown to demonstrate neuroprotective properties and hence may have additional benefits in conditions such as traumatic brain injury.^54^

We had previously identified a novel pathway to modulate ICP in an animal model.^7^ This preclinical work has now been translated to a population of patients with raised ICP due to IIH. The data demonstrated the efficacy of exenatide to significantly lower ICP. A new efficacious therapy is a clear unmet patient need,^24^ and this is the first trial of a new drug treatment for IIH that significantly reduces ICP. The data presented here support further evaluation of exenatide in a large randomized controlled trial, powered to evaluate clinically relevant outcome measures.

## Supplementary Material

awad003_Supplementary_Data

## References

[1] Drucker DJ . Biological actions and therapeutic potential of the glucagon-like peptides. Gastroenterology. 2002;122:531–544. 11832466 10.1053/gast.2002.31068

[2] Alvarez E , Roncero I, Chowen JA, Thorens B, Blázquez E. Expression of the glucagon-like peptide-1 receptor gene in rat brain. J Neurochem. 1996;66:920–927. 8769850 10.1046/j.1471-4159.1996.66030920.x

[3] Carr RD , Larsen MO, Jelic K, et al Secretion and dipeptidyl peptidase-4-mediated metabolism of incretin hormones after a mixed meal or glucose ingestion in obese compared to lean, nondiabetic men. Comparative study. J Clin Endocrinol Metab. 2010;95:872–878. 20008019 10.1210/jc.2009-2054

[4] Rubino D , Abrahamsson N, Davies M, et al Effect of continued weekly subcutaneous semaglutide vs placebo on weight loss maintenance in adults with overweight or obesity: The STEP 4 randomized clinical trial. JAMA. 2021;325:1414–1425. 33755728 10.1001/jama.2021.3224 PMC7988425

[5] Gutzwiller JP , Tschopp S, Bock A, et al Glucagon-like peptide 1 induces natriuresis in healthy subjects and in insulin-resistant obese men. Clinical trial. J Clin Endocrinol Metab. 2004;89:3055–3061. 15181098 10.1210/jc.2003-031403

[6] von Websky K , Reichetzeder C, Hocher B. Physiology and pathophysiology of incretins in the kidney. Review. Curr Opin Nephrol Hypertens. 2014;23:54–60. 24257158 10.1097/01.mnh.0000437542.77175.a0

[7] Botfield HF , Uldall MS, Westgate CSJ, et al A glucagon-like peptide-1 receptor agonist reduces intracranial pressure in a rat model of hydrocephalus. Sci Transl Med. 2017;9:eaan0972. 10.1126/scitranslmed.aan0972 28835515

[8] Ast J , Arvaniti A, Fine NHF, et al Super-resolution microscopy compatible fluorescent probes reveal endogenous glucagon-like peptide-1 receptor distribution and dynamics. Nat Commun. 2020;11:467. 31980626 10.1038/s41467-020-14309-w PMC6981144

[9] Scotton WJ , Botfield HF, Westgate CS, et al Topiramate is more effective than acetazolamide at lowering intracranial pressure. Cephalalgia. 2019;39:209–218. 29898611 10.1177/0333102418776455 PMC6376637

[10] Mollan SP , Grech O, Alimajstorovic Z, Wakerley BR, Sinclair AJ. New horizons for idiopathic intracranial hypertension: Advances and challenges. Br Med Bull. 2020;136:118–126. 33200788 10.1093/bmb/ldaa034

[11] Mollan SP , Tahrani AA, Sinclair AJ. The potentially modifiable risk factor in idiopathic intracranial hypertension: Body weight. Neurol Clin Pract. 2021;11:e504–e507. 34484948 10.1212/CPJ.0000000000001063 PMC8382420

[12] Mollan SP , Mitchell JL, Ottridge RS, et al Effectiveness of bariatric surgery vs community weight management intervention for the treatment of idiopathic intracranial hypertension: A randomized clinical trial. JAMA Neurol. 2021;78:678–686. 33900360 10.1001/jamaneurol.2021.0659 PMC8077040

[13] Sinclair AJ , Burdon MA, Nightingale PG, et al Low energy diet and intracranial pressure in women with idiopathic intracranial hypertension: Prospective cohort study. BMJ. 2010;341:c2701. 20610512 10.1136/bmj.c2701 PMC2898925

[14] Mollan SP , Aguiar M, Evison F, Frew E, Sinclair AJ. The expanding burden of idiopathic intracranial hypertension. Eye. 2019;33:478–485. 30356129 10.1038/s41433-018-0238-5 PMC6460708

[15] Adderley NJ , Subramanian A, Nirantharakumar K, et al Association between idiopathic intracranial hypertension and risk of cardiovascular diseases in women in the United Kingdom. JAMA Neurol. 2019;76:1088–1098. 31282950 10.1001/jamaneurol.2019.1812 PMC6618853

[16] Mollan SP , Mytton J, Tsermoulas G, Sinclair AJ. Idiopathic intracranial hypertension: Evaluation of admissions and emergency readmissions through the hospital episode statistic dataset between 2002–2020. Life (Basel). 2021;11:417. 34063037 10.3390/life11050417 PMC8148005

[17] McCluskey G , Doherty-Allan R, McCarron P, et al Meta-analysis and systematic review of population-based epidemiological studies in idiopathic intracranial hypertension. Eur J Neurol. 2018;25:1218–1227. 29953685 10.1111/ene.13739

[18] Wall M , George D. Idiopathic intracranial hypertension. A prospective study of 50 patients. Brain. 1991;114:155–180. 1998880

[19] Corbett JJ , Savino PJ, Thompson HS, et al Visual loss in pseudotumor cerebri. Follow-up of 57 patients from five to 41 years and a profile of 14 patients with permanent severe visual loss. Arch Neurol. 1982;39:461–474. 7103794 10.1001/archneur.1982.00510200003001

[20] Mulla Y , Markey KA, Woolley RL, Patel S, Mollan SP, Sinclair AJ. Headache determines quality of life in idiopathic intracranial hypertension. J Headache Pain. 2015;16:521. 25982204 10.1186/s10194-015-0521-9 PMC4436432

[21] Mollan SP , Wakerley BR, Alimajstorovic Z, et al Intracranial pressure directly predicts headache morbidity in idiopathic intracranial hypertension. J Headache Pain. 2021;22:118. 34620087 10.1186/s10194-021-01321-8 PMC8499560

[22] Grech O , Clouter A, Mitchell JL, et al Cognitive performance in idiopathic intracranial hypertension and relevance of intracranial pressure. Brain Commun. 2021;3:fcab202. 10.1093/braincomms/fcab202 PMC8421706 34704028

[23] Mollan SP , Davies B, Silver NC, et al Idiopathic intracranial hypertension: Consensus guidelines on management. J Neurol Neurosurg Psychiatry. 2018;89:1088–1100. 29903905 10.1136/jnnp-2017-317440 PMC6166610

[24] Mollan S , Hemmings K, Herd CP, Denton A, Williamson S, Sinclair AJ. What are the research priorities for idiopathic intracranial hypertension? A priority setting partnership between patients and healthcare professionals. BMJ Open. 2019;9:e026573. 10.1136/bmjopen-2018-026573 PMC6429891 30878991

[25] Friedman DI , Liu GT, Digre KB. Revised diagnostic criteria for the pseudotumor cerebri syndrome in adults and children. Neurology. 23 2013;81:1159–1165. 23966248 10.1212/WNL.0b013e3182a55f17

[26] Kosinski M , Bayliss MS, Bjorner JB, et al A six-item short-form survey for measuring headache impact: The HIT-6. Qual Life Res. 2003;12:963–974. 14651415 10.1023/a:1026119331193

[27] Hays RD , Morales LS. The RAND-36 measure of health-related quality of life. Ann Med. 2009;33:350–357. 10.3109/07853890109002089 11491194

[28] Mitchell JL , Buckham R, Lyons H, et al Evaluation of diurnal and postural intracranial pressure employing telemetric monitoring in idiopathic intracranial hypertension. Fluids Barriers CNS. 2022;19:85. 36320018 10.1186/s12987-022-00384-2 PMC9628104

[29] Tomlinson JW , Finney J, Gay C, Hughes BA, Hughes SV, Stewart PM. Impaired glucose tolerance and insulin resistance are associated with increased adipose 11beta-hydroxysteroid dehydrogenase type 1 expression and elevated hepatic 5alpha-reductase activity. Diabetes. 2008;57:2652–2660. 18633104 10.2337/db08-0495 PMC2551674

[30] Stewart PM , Boulton A, Kumar S, Clark PM, Shackleton CH. Cortisol metabolism in human obesity: Impaired cortisone–>cortisol conversion in subjects with central adiposity. J Clin Endocrinol Metab. 1999;84:1022–1027. 10084590 10.1210/jcem.84.3.5538

[31] Levy JC , Matthews DR, Hermans MP. Correct homeostasis model assessment (HOMA) evaluation uses the computer program. Diabetes Care. 1998;21:2191–2192. 9839117 10.2337/diacare.21.12.2191

[32] Li Y , Vaughan KL, Tweedie D, et al Pharmacokinetics of exenatide in nonhuman primates following its administration in the form of sustained-release PT320 and bydureon. Sci Rep. 2019;9:17208. 31748513 10.1038/s41598-019-53356-2 PMC6868133

[33] Peng H , Want LL, Aroda VR. Safety and tolerability of glucagon-like peptide-1 receptor agonists utilizing data from the exenatide clinical trial development program. Curr Diab Rep. 2016;16:44. 27037706 10.1007/s11892-016-0728-4

[34] Fineman MS , Mace KF, Diamant M, et al Clinical relevance of anti-exenatide antibodies: Safety, efficacy and cross-reactivity with long-term treatment. Diabetes Obes Metab. 2012;14:546–554. 22236356 10.1111/j.1463-1326.2012.01561.x

[35] Chen S , Yu SJ, Li Y, et al Post-treatment with PT302, a long-acting exendin-4 sustained release formulation, reduces dopaminergic neurodegeneration in a 6-hydroxydopamine rat model of Parkinson’s disease. Sci Rep. 2018;8:10722. 30013201 10.1038/s41598-018-28449-z PMC6048117

[36] Ogashiwa M , Takeuchi K. [Intracranial pressure changes during sleep in man]. No To Shinkei. 1983;35:123–129. 6682671

[37] Mollan SP , Grech O, Sinclair AJ. Headache attributed to idiopathic intracranial hypertension and persistent post-idiopathic intracranial hypertension headache: A narrative review. Headache. 2021;61:808–816. 34106464 10.1111/head.14125

[38] Mollan SP , Sinclair AJ. Outcomes measures in idiopathic intracranial hypertension. Expert Rev Neurother. 2021;21:687–700. 34047224 10.1080/14737175.2021.1931127

[39] Mitchell JL , Mollan SP, Tsermoulas G, Sinclair AJ. Telemetric monitoring in idiopathic intracranial hypertension demonstrates intracranial pressure in a case with sight-threatening disease. Acta Neurochir (Wien). 2021;163:725–731. 33411042 10.1007/s00701-020-04640-y

[40] Ball AK , Howman A, Wheatley K, et al A randomised controlled trial of treatment for idiopathic intracranial hypertension. J Neurol. 2010;258:874–881. 21161260 10.1007/s00415-010-5861-4

[41] Wall M , McDermott MP, Kieburtz KD, et al Effect of acetazolamide on visual function in patients with idiopathic intracranial hypertension and mild visual loss. JAMA. 2014;311:1641–1624. 24756514 10.1001/jama.2014.3312 PMC4362615

[42] Markey K , Mitchell J, Botfield H, et al 11β-Hydroxysteroid dehydrogenase type 1 inhibition in idiopathic intracranial hypertension: A double-blind randomized controlled trial. Brain Commun. 2020;2:874. 10.1093/braincomms/fcz050 PMC7425517 32954315

[43] Mollan SP , Hoffmann J, Sinclair AJ. Advances in the understanding of headache in idiopathic intracranial hypertension. Curr Opin Neurol. 2019;32:92–98. 30547900 10.1097/WCO.0000000000000651 PMC6343949

[44] Ottridge R , Mollan SP, Botfield H, et al Randomised controlled trial of bariatric surgery versus a community weight loss programme for the sustained treatment of idiopathic intracranial hypertension: The idiopathic intracranial hypertension weight trial (IIH:WT) protocol. BMJ Open. 2017;7:e017426. 10.1136/bmjopen-2017-017426 PMC5623580 28963303

[45] Tepper S , Ashina M, Reuter U, et al Safety and efficacy of erenumab for preventive treatment of chronic migraine: A randomised, double-blind, placebo-controlled phase 2 trial. Clinical trial, phase II. Lancet Neurol. 2017;16:425–434. 28460892 10.1016/S1474-4422(17)30083-2

[46] Silberstein SD , Dodick DW, Bigal ME, et al Fremanezumab for the preventive treatment of chronic migraine. Clinical trial, phase III. N Engl J Med. 30 2017;377:2113–2122. 29171818 10.1056/NEJMoa1709038

[47] Beck RW , Maguire MG, Bressler NM, Glassman AR, Lindblad AS, Ferris FL. Visual acuity as an outcome measure in clinical trials of retinal diseases. Ophthalmology. 2007;114:1804–1809. 17908590 10.1016/j.ophtha.2007.06.047

[48] Buse JB , Drucker DJ, Taylor KL, et al DURATION-1: Exenatide once weekly produces sustained glycemic control and weight loss over 52 weeks. Multicenter study. Diabetes Care. 2010;33:1255–1261. 20215461 10.2337/dc09-1914 PMC2875434

[49] Kolterman OG , Kim DD, Shen L, et al Pharmacokinetics, pharmacodynamics, and safety of exenatide in patients with type 2 diabetes mellitus. Am J Health Syst Pharm. 2005;62:173–181. 15700891 10.1093/ajhp/62.2.173

[50] MacConell L , Gurney K, Malloy J, Zhou M, Kolterman O. Safety and tolerability of exenatide once weekly in patients with type 2 diabetes: An integrated analysis of 4,328 patients. Diabetes Metab Syndr Obes. 2015;8:241–253. 26056482 10.2147/DMSO.S77290 PMC4445788

[51] Kothare PA , Linnebjerg H, Isaka Y, et al Pharmacokinetics, pharmacodynamics, tolerability, and safety of exenatide in Japanese patients with type 2 diabetes mellitus. J Clin Pharmacol. 2008;48:1389–1399. 19047364 10.1177/0091270008323750

[52] Westgate CS , Botfield HF, Alimajstorovic Z, et al Systemic and adipocyte transcriptional and metabolic dysregulation in idiopathic intracranial hypertension. JCI Insight. 2021;6: e145346. 10.1172/jci.insight.145346 PMC8262372 33848268

[53] Mitchell JL , Mollan SP, Vijay V, Sinclair AJ. Novel advances in monitoring and therapeutic approaches in idiopathic intracranial hypertension. Currt Opin Neurol. 2019;32:422–431. 10.1097/WCO.0000000000000690 PMC6522204 30865008

[54] Erbil D , Eren CY, Demirel C, Kucuker MU, Solaroglu I, Eser HY. GLP-1’s role in neuroprotection: A systematic review. Brain Inj. 2019;33:734–819. 30938196 10.1080/02699052.2019.1587000

