# Supplementary material for: The effect of GLP-1RA exenatide on idiopathic intracranial hypertension: a randomized clinical trial
Source: Brain. 2023 Mar 13;146(5):1821–30. doi: 10.1093/brain/awad003 (PMC10151178; doi:10.1093/brain/awad003)
Supplement: awad003_Supplementary_Data [file awad003_supplementary_data.pdf]

The effect of GLP-1RA exenatide on Idiopathic Intracranial Hypertension: Randomised Clinical Trial

Supplementary tables and figures.

Supplementary Table 1 Inclusion and exclusion criteria

**Inclusion Criteria**
Female IIH patients aged between 18 and 60 years, diagnosed according to the modified Dandy criteria who have active disease (papilloedema [Frisen grade ≥ 1], significantly raised ICP > 25cmH_2O) and no evidence of venous sinus thrombosis (magnetic resonance imaging (MRI) or computerised tomography (CT) imaging and venography as noted at diagnosis).
Able to give informed consent.
**Exclusion Criteria**
Age less than 18 or older than 60 years.
Pregnant or trying to conceive.
Significant co-morbidity; such that in the opinion of the investigator it would not be in the participant's best interest to participate in the trial.
Addison's or Cushing's disease.
Functioning CSF shunt/stent or optic nerve sheath fenestration.
Currently using GLP-1 agonist or DPP-4 inhibitor.
Surgical contra-indication.
Concomitant therapy with acetazolamide, topiramate or diuretics (this can be discontinued 1 month prior to enrolment).
Inability to give informed consent e.g. due to cognitive impairment.

Supplementary Table 2 Schedule of events

	Enrolment	Surgical implant	Baseline						Treatment period		
**Week**	-4 (minimum)		1						2	8	12
**Day**			1					2	14	56	84
**Hour**			pre-dose	0	2.5	6	11	24			
**Identification, trial and eligibility discussion, provision of PIS**	X										
**Eligibility**	X										
**Informed consent signature**	X										
**Medical History**	X		X								
**HCG testing**	X		X								
**Provision of 4-week headache diary**	X									X	
**Physical Examination and clinical measurements**	X		X								X
**Insertion of ICP monitor**		X									
**Randomisation**			X								
**Telemetric pressure monitoring (ICP monitoring)**			X[2]		X[3]			X[3]			X[3]
**LogMAR Visual Acuity**			X								X
**Intraocular pressure**			X								X
**Perimetric Mean Deviation (PMD – HVF)**			X								X
**Optical Coherence Tomography RNFL**			X								X
**Headache diary review**			X								X
**BMI**			X								X
**Blood pressure and heart rate**	x	x	x								x
**Questionnaires: HIT-6 and SF-36**			X								X
**Blood sampling – Biochemistry and ADA**			X						X		X
**Blood sampling PK**			X	X	X	X	X	X		X	X
**DEXA**			X								X

Supplementary Table 3 Baseline characteristics

	**All Mean (SD)**	**Exenatide Mean (SD)**	**Placebo Mean (SD)**
Number (n)	15	7	8
Age	28 (9)	28 (13)	28 (6)
BMI (kg/m2)	38.1 (6.2)	37.6 (7.9)	38.6 (4.7)
ICP (supine) mmHg	23.5 (3.9)	22.3 (3.6)	24.6 (4.1)
ICP (LP position) cm CSF	32.2 (5.6)	30.7 (6.7)	33.5 (5.6)
Frisen Grade (Worst eye)* median(IQR)	2 (1)	2 (1)	2.5 (1)
	**Median (IQR)**	**Median (IQR)**	**Median (IQR)**
Duration of IIH at enrolment (months)	7 (35.5)	4 (15.5)	18.5 (55.3)
Time from surgery to baseline Time from surgery to baseline	10 (16.5)10 (16.5)	4 (3.5)	18.5 (19.5)

Supplementary Table 4 Secondary and exploratory outcomes

	Baseline mean (SD), n	12 weeks mean (SD), n	Difference baseline to 12 weeks mean (SD); 95%CI, p	Difference between arms at 12 weeks mean (SE); 95%CI, p Hierarchical regression
**Monthly headache days**				
Exenatide	21.6 (5.2), n=7	13.9 (7.2), n=7	-7.7 (9.2); (-16.3, 0.8), p=0.069	5.1 (3.9); (-2.4, 12.7), p=0.184
Placebo	10.3 (8.5), n=8	8.8 (8.0), n=8	-1.5 (4.8); (-5.5, 2.5), p=0.404	
**Monthly analgesic frequency**				
Exenatide	7.9 (4.5), n=7	7.0 (5.7), n=7	-0.9 (5.2); (-5.7, 4.0), p=0.680	1.1 (2.5); (-3.8, 6.0), p=0.648
Placebo	3.4 (2.8), n=7	5.9 (5.2), n=7	2.4 (5.1); (-2.3, 7.1), p=0.254	
**LogMar visual acuity**				
Exenatide	0.0 (0.05), n=7	-0.1 (0.07), n=7	-0.1 (0.04); (-0.1, 0.0), p=0.004	-0.1 (0.05); (-0.2, -0.1), p=0.036
Placebo	0.0 (0.14), n=8	0.0 (0.12), n=8	0.0 (0.14); (-0.1, 0.1), p=0.921	
**Perimetric mean deviation worst eye dB (HVF 24-2 sita standard)**				
Exenatide	-0.6 (1.0), n=7	-1.0 (0.9), n=7	-0.3 (1.1); (-1.4, 0.7), p=0.472	1.0 (0.8); (-0.5, 2.5), p=0.188
Placebo	-2.7 (1.9), n=8	-2.0 (1.6), n=8	0.7 (0.7); (0.1, 1.3), p=0.020	
**Intraocular pressure**				
Exenatide	18.0 (2.0), n=7	16.9 (1.7), n=7	-1.2 (2.8); (-3.8, 1.4), p=0.306	-0.1 (1.1); (-2.3, 2.1), p=0.910
Placebo	16.7 (2.6), n=8	16.9 (1.9), n=7	0.5 (1.3); (-0.7, 1.7), p=0.375	
**Optical Coherence Tomography RNFL worst eye (µm)**				
Exenatide	153 (58.9), n=6	132 (34.0), n=6	-21.0 (28.8); (-51.2, 9.2), p=0.134	-40.2 (47.2); (-133.0, 52.4), p=0.396
Placebo	183 (100.0), n=8	172 (114.0), n=8	-10.8 (88.0); (-84.3, 62.8), p=0.740	
**Quality of Life (SF-36) PCS summary**				
Exenatide	49.7 (20.3), n=7	53.8 (23.4), n=7	4.1 (7.4); (-2.7, 10.9), p=0.191	-5.7 (9.5); (-24.3, 12.9), p=0.550
Placebo	57.8 (16.9), n=8	59.5 (11.8), n=8	1.7 (9.5); (-6.2, 9.6), p=0.632	
**Quality of Life (SF-36) MCS summary**				
Exenatide	43.4 (23.3), n=7	44.8 (24.2), n=7	1.4 (8.3); (-7.3, 10.1), p=0.692	-2.3 (10.3); (-22.5, 18.0), p=0.826
Placebo	46.6 (17.2), n=8	46.9 (10.5), n=8	0.5 (16.3); (-14.6, 15.5), p=0.940	
**BMI (kg/m2**				
Exenatide	37.6 (7.9), n=7	37.5 (7.4), n=7	-0.1 (0.8); (-0.8, 0.7), p=0.851	-0.6 (3.3); (-7.0, 5.8), p=0.854
Placebo	38.6 (4.7), n=8	38.1 (4.9), n=8	-0.5 (1.3); (-1.6, 0.6), p=0.336	
**Mean arterial pressure (mmHg)**				unpaired t-Test
Exenatide	92.3 (10.8), n=7	85.8 (6.2), n=7	-6.5 (12.9); (-18.4, 5.4), p=0.23	-6.7 (3.6); (-1.1, 14.5), p=0.088
Placebo	89.6 (6.2), n=8	92.4 (5.2), n=8	2.8 (6.4); (-2.6, 8.1), p=0.26	

Supplementary Table 5 Headache severity

	Baseline			12 weeks		
**Headache severity (VRS 0-10) Category**	**Exenatide**	**Placebo**	**chi-squared p**	**Exenatide**	**Placebo**	**chi-squared p**
Mild, n (%)	0 (0%)	3 (38%)	0.133	0 (0%)	4 (50%)	0.084
Moderate	6 (86%)	5 (62%)		6 (86%)	3 (38%)	
Severe	1 (14%)	0 (0%)		1 (14%)	1 (13%)	

Supplementary Table 6. Headache disability

Headache disability (HIT-6) Category	Baseline n (%)			12 weeks n (%)		
	Exenatide	Placebo	chi-squared p	Exenatide	Placebo	chi-squared p
Little-to-no impact	0 (0%)	2 (25%)	0.218	0 (0%)	3 (38%)	0.088
Moderate	0 (0%)	1 (13%)		1 (14%)	0 (0%)	
Severe	5 (71%)	2 (25%)		4 (57%)	3 (38%)	
Substantial	2 (29%)	3 (38%)		0 (0%)	2 (25%)	
Missing				2 (29%)		

Supplementary Table 7 Blood test results

	Baseline mean (SD), n	12 weeks mean (SD), n	Difference baseline to 12 weeks mean (SD); 95%CI, p	Difference between arms at 12 weeks mean (SE); 95%CI, p (Hierarchical regression)
**Creatinine (μmol/L)**				
Exenatide	67.6 (9.5), n=7	72.7 (3.5), n=7	5.1 (7.4); (-1.7, 12.0), p=0.117	5.6 (4.2); (-2.7, 13.9), p=0.186
Placebo	66.4 (8.1), n=8	67.1 (9.7), n=8	0.8 (7.0); (-5.1, 6.6), p=0.770	
**Alanine transaminase (IU/L)**				
Exenatide	27.2 (13.9), n=6	24.9 (17.2), n=7	-0.2 (7.9); (-8.4, 8.1), p=0.961	8.0 (6.5); (-4.7, 20.7), p=0.218
Placebo	21.3 (11.7), n=8	16.8 (5.4), n=8	-4.4 (7.3); (-10.5, 1.7), p=0.134	
**High density lipoprotein (mmol/L)**				
Exenatide	1.3 (0.4), n=7	1.2 (0.2), n=7	0.0 (0.3); (-0.3, 0.2), p=0.710	-0.2 (0.1); (-0.5, 0.0), p=0.088
Placebo	1.5 (0.2), n=8	1.5 (0.3), n=8	0.0 (0.2); (-0.2, 0.2), p=0.866	
**Cholesterol (mmol/L)**				
Exenatide	4.5 (0.8), n=7	4.7 (1.0), n=7	0.2 (0.6); (-0.4, 0.7), p=0.472	0.0 (0.6); (-1.1, 1.1), p=0.964
Placebo	4.8 (1.0), n=8	4.7 (1.4), n=8	-0.1 (0.7); (-0.6, 0.5), p=0.715	
**Triglycerides (mmol/L)**				
Exenatide	1.3 (0.6), n=7	1.3 (0.5), n=7	0.1 (0.5); (-0.4, 0.6), p=0.680	0.2 (0.2); (-0.3, 0.6), p=0.488
Placebo	1.1 (0.2), n=8	1.2 (0.4), n=7	0.0 (0.4); (-0.3, 0.4), p=0.782	
**HbA1C (mmol/mol)**				
Exenatide	35.4 (2.7), n=5	36.3 (2.9), n=7	1.0 (1.4); (-0.8, 2.8), p=0.189	1.8 (1.6); (-1.4, 5.0), p=0.278
Placebo	35.0 (3.9), n=6	34.5 (3.0), n=8	0.7 (2.7); (-2.2, 3.5), p=0.576	

Supplementary Table 8 Adverse events

	Number of events	Arm Exenatide n	Placebo n	Severity	Relatedness	Description
**Adverse Events**						
Nausea	3	3	0	Moderate	Related	Nausea requiring treatment on baseline visit
Nausea	4	4	0	Mild	Related	Mild transient nausea
Minor wound infection	3	1	2	Mild	Unrelated	Participant continued in trial
Post-Operative facial swelling	1	0	1	Mild	Unrelated	Participant continued in trial
**Serious Adverse Events**						
Thyrotoxicosis	1	0	1	Moderate	Unrelated	Participant continued in trial
Withdrawals						
Withdrawals	1	1	0			Pre-randomisation

Supplementary Table 9 Exenatide serum concentration

Timepoint	Day 0 20 µg Exenatide pg/ml (mean (SD)) n=7	Day 14 10 µg Exenatide BD pg/ml (mean (SD)) n=4	Day 84 10 µg Exenatide BD pg/ml (mean (SD)) n=4
0	44.4 (12.0)	81.3 (54.7)	91.6 (61.8)
2.5	575.4 (501.6)	380.7 (180.4)	205.4 (83.2
6	180.9 (84.9)		
11	67.3 (22.5)		
22	44.6 (16.8)		
24	335.8 (106.5)		

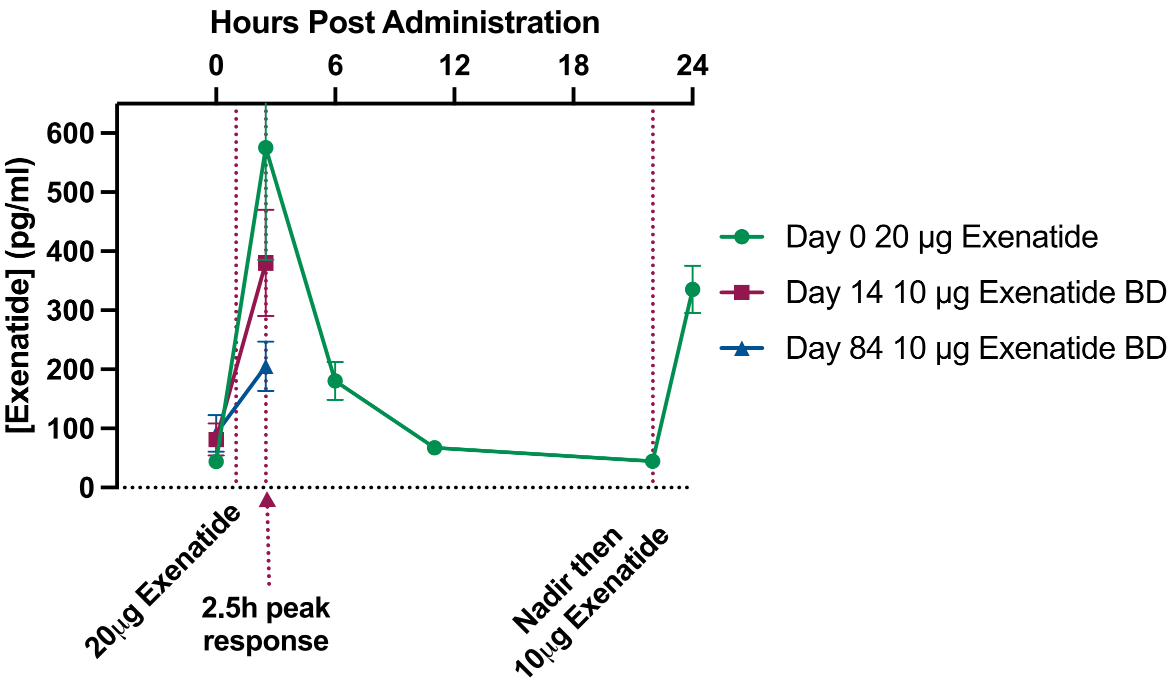

Supplementary Figure 1 Exenatide serum concentration (mean and SEM). Serum exenatide levels were measured after single bolus administration of exenatide 20µg on day 0 and following 10µg dosing on day 14 and 84.

Supplementary Table 10 Insulin and glucose serum concentration

	Baseline mean (SD), n	12 weeks mean (SD), n	Difference baseline to 12 weeks T-test, mean (SD); 95%CI, p	Difference between arms at 12 weeks T-test, mean (SE); 95%CI, p
**Insulin**				
Exenatide	98.0 (49.4), 6	163.2 (114.6), 6	59.6 (91.75); (-36.7, 155.9) p=0.17	88.6 (51.2); -23.0, 200.3) p=0.11
Placebo	70.0 (42.3), 8	74.5 (77.8), 8	4.6 (59.1); (-44.9, 54.0) p=0.83	
**Glucose**				
Exenatide	3.2 (0.7), 5	3.6 (0.5), 5	0.5 (1.0); (-0.8, 1.7) p=0.36	0.26 (0.26); (-0.3, 0.8) p=0.34
Placebo	3.2 (0.3), 8	3.4 (0.4), 8	0.1 (0.2); (-0.05, 0.3) p=0.13	

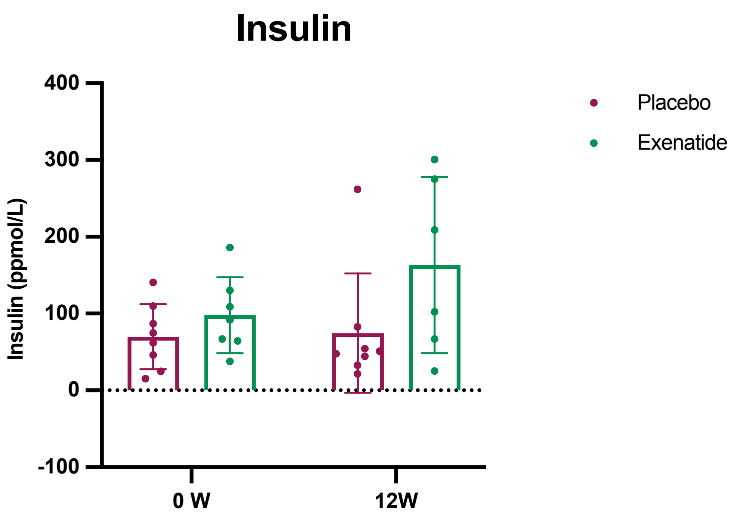

Supplementary Figure 2 Insulin and glucose serum concentration. Blood insulin and glucose concentrations were measured at baseline and 12 weeks.

Supplementary table 11 ICP monitoring, 2.5-hour time course

Time	Baseline ICP (mmHg) mean (SD)	At time point ICP (mmHg) mean (SD)	Difference baseline to time point mean (SD); 95%CI, p	Difference between arms at time point mean (SE); 95%CI, p (Hierarchical regression)[a]
**0-30 mins**				
Exenatide	22.3 (3.6)	23.3 (4.1)	1.0 (1.3); (-0.2, 2.2), p=0.089	-2.3 (2.1); (-6.3, 1.8), p=0.274
Placebo	24.6 (4.1)	25.4 (4.0)	1.6 (2.0); (-.03, 3.4), p=0.080	
**30-60 mins**				
Exenatide	22.3 (3.6)	23.7 (4.9)	1.4 (2.1); (-0.5, 3.3), p=0.125	-3.4 (2.1); (-7.5, 0.7), p=0.106
Placebo	24.6 (4.1)	26.3 (4.8)	2.5 (2.8); (-0.1, 5.1), p=0.055	
**60-90 mins**				
Exenatide	22.3 (3.6)	22.6 (4.7)	0.3 (1.9); (-1.4, 2.1), p=0.637	-3.4 (2.1); (-7.5, 0.7), p=0.102
Placebo	24.6 (4.1)	25.3 (2.8)	1.5 (2.6); (-0.9, 3.9), p=0.174	
**90-120 mins**				
Exenatide	22.3 (3.6)	22.0 (3.8)	-0.4 (2.8); (-3.3, 2.6), p=0.769	-4.6 (2.1); (-8.8, -0.5), p=0.028
Placebo	24.6 (4.1)	25.7 (2.4)	1.8 (2.7); (-0.6, 4.3), p=0.116	
**120-150 mins**				
Exenatide	22.3 (3.6)	21.8 (3.4)	-0.5 (1.9); (-2.3, 1.2), p=0.485	-4.2 (2.1); (-8.3, -0.2), p=0.042
Placebo	24.6 (4.1)	26.0 (3.4)	1.4 (1.8); (-0.1, 2.9), p=0.060	

[a]Due to the nature of hierarchical analysis, there is slight variation in values compared to those of the primary outcomes, however data included at timepoints is identical.

Supplementary Table 12 Overnight ICP monitoring

Time	At time point ICP (mmHg) mean (SD)	Difference between arms at time point mean (SE); 95%CI, p (Hierarchical regression)
**Midnight (baseline) – 01:00**		
Exenatide	16.1 (3.5)	-2.5 (2.3); (-7.1, 2.1), p=0.284
Placebo	18.2 (5.7)	
**01:00 – 02:00**		
Exenatide	14.7 (3.2)	-4.0 (2.3); (-8.4, 0.5), p=0.082
Placebo	18.7 (4.0)	
**02:00 – 03:00**		
Exenatide	15.2 (3.8)	-1.9 (2.3); (-6.4, 2.6), p=0.406
Placebo	17.1 (3.9)	
**03:00 – 04:00**		
Exenatide	15.4 (2.2)	-2.9 (2.3); (-7.4, 1.6), p=0.206
Placebo	18.3 (4.4)	
**04:00 – 05:00**		
Exenatide	14.5 (4.8)	-5.6 (2.3); (-10.1, -1.0), p=0.018
Placebo	20.7 (4.1)	
**05:00 – 06:00**		
Exenatide	16.1 (3.2)	-4.2 (2.5); (-9.1, 0.6), p=0.086
Placebo	20.1 (3.1)	
**06:00 – 07:00**		
Exenatide	16.2 (6.0)	-5.0 (2.5); (-9.9, -0.2), p=0.040
Placebo	22.1 (4.1)	

Supplementary Table 13 DEXA body composition analysis

	Baseline mean (SD), n	12 weeks mean (SD), n	Difference baseline to 12 weeks T-test, mean (SD); 95%CI, p	Difference between arms at 12 weeks T-test, mean (SE); 95%CI, p
**Fat % total**				
Exenatide	51.4 (3.1), n=7	51.2 (3.3), n=7	-0.17 (1.1); (-1.2, 0.9), p=0.70	0.50 (1.8); (-3.4, 4.4), p=0.78
Placebo	51.9 (3.9), n=8	50.7 (3.6), n=8	-1.2 (1.6); (-2.6, 0.08), p=0.06	
**Fat mass**				
Exenatide	51.0 (16.5), n=7	51.2 (16.8), n=7	0.15 (0.14); (-1.1, 1.4), p=0.78	2.5 (7.0); (-12.7, 17.7), p=0.73
Placebo	50.9 (11.1), n=8	48.6 (10.1), n=8	-2.3 (3.2); (-5.0, 0.4), p=0.086	
**Lean mass**				
Exenatide	47.0 (10.0), n=7	47.4 (10.1), n=7	0.4 (1.0); (-0.5, 1.4), p=0.31	0.8 (4.2); (-8.2, 9.7), p=0.86
Placebo	46.3 (5.4), n=8	46.6 (5.7), n=8	0.29 (1.5); (-0.9, 1.5), p=0.60	
**DEXA A/G ratio**				
Exenatide	1.1 (0.08), n=7	1.1 (0.08), n=7	-0.009 (0.04); (-0.04, 0.03) p=0.57	0.05 (0.04); (-0.05, 0.1), p=0.28
Placebo	1.1 (0.08), n=8	1.1 (0.09), n=8	0.001 (0.03); (-0.02, 0.02), p=0.90	
